# Supplementary material for: An Interpretable Machine-Learning Algorithm to Predict Disordered Protein Phase Separation Based on Biophysical Interactions
Source: Biomolecules. 2022 Aug 17;12(8):1131. doi: 10.3390/biom12081131 (PMC9405563; doi:10.3390/biom12081131)
Supplement: Supplementary file 1 [file biomolecules-12-01131-s001.zip › LLPhyScore_Supplementary_Material.pdf]

Supplementary Material for:

An interpretable machine learning algorithm to predict disordered protein phase separation based on biophysical interactions

Hao Cai[1,+], Robert M. Vernon[1,++], Julie D. Forman-Kay[1,2]

[1]Molecular Medicine Program, Hospital for Sick Children, Toronto, Ontario M5G 0A4, Canada
[2]Department of Biochemistry, University of Toronto, Toronto, Ontario, M5S 1A8, Canada
[+]Current Address: Shopify Inc., 150 Elgin Street 8th Floor, Ottawa, ON K2P 1L4, Canada
[++]Current Address: Amgen Inc. Research and Development, Burnaby, BC V5A 1V7, Canada

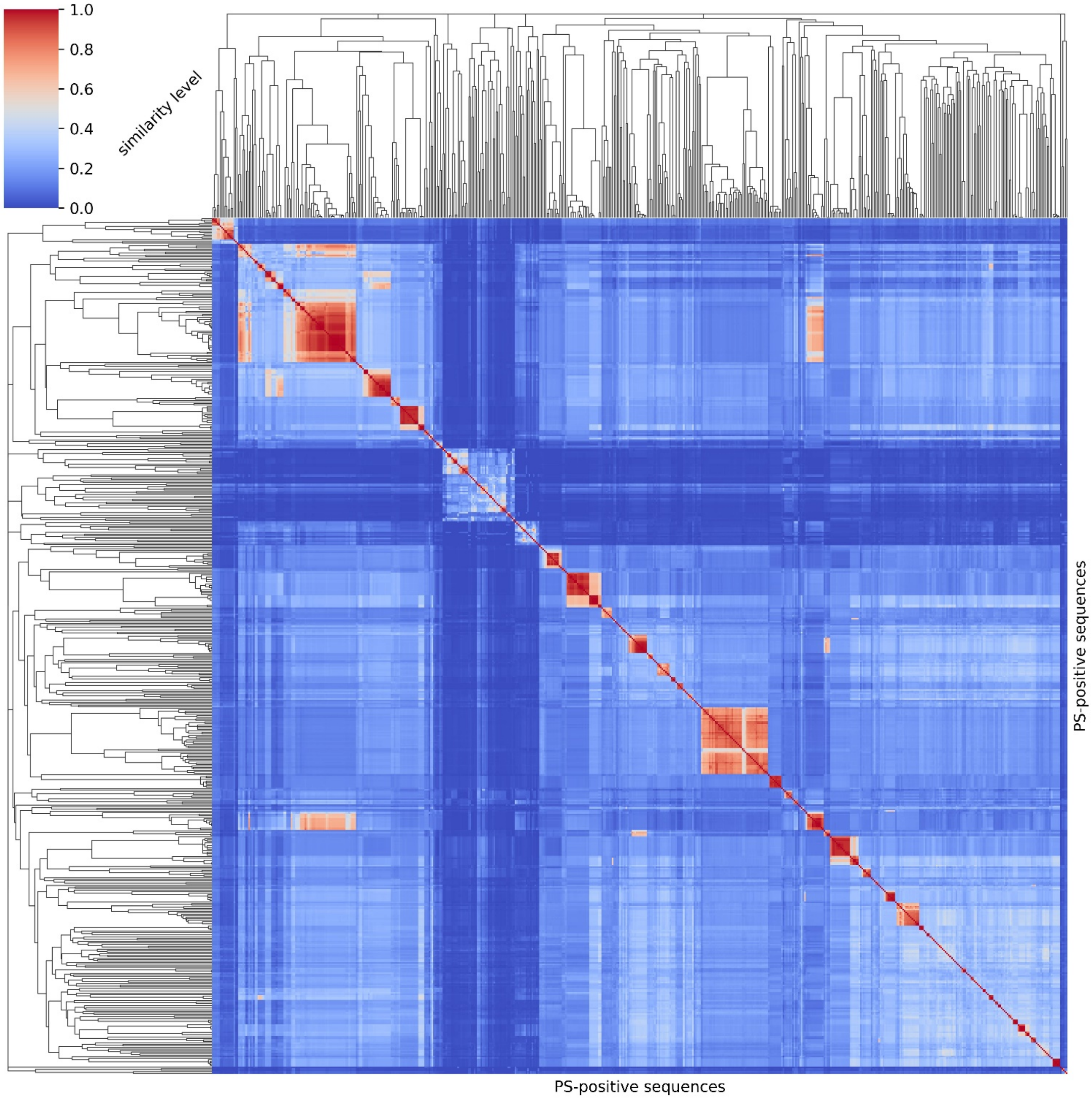

Figure S1. Sequence clustering map of PS-positive sequences. Left and top: clustering dendrograms showing the pairwise similarity level (0 - 1.0) for all sequences. Middle: heatmap of similarity levels showing that inter-cluster sequences have pairwise similarity higher than 0.5 in general.

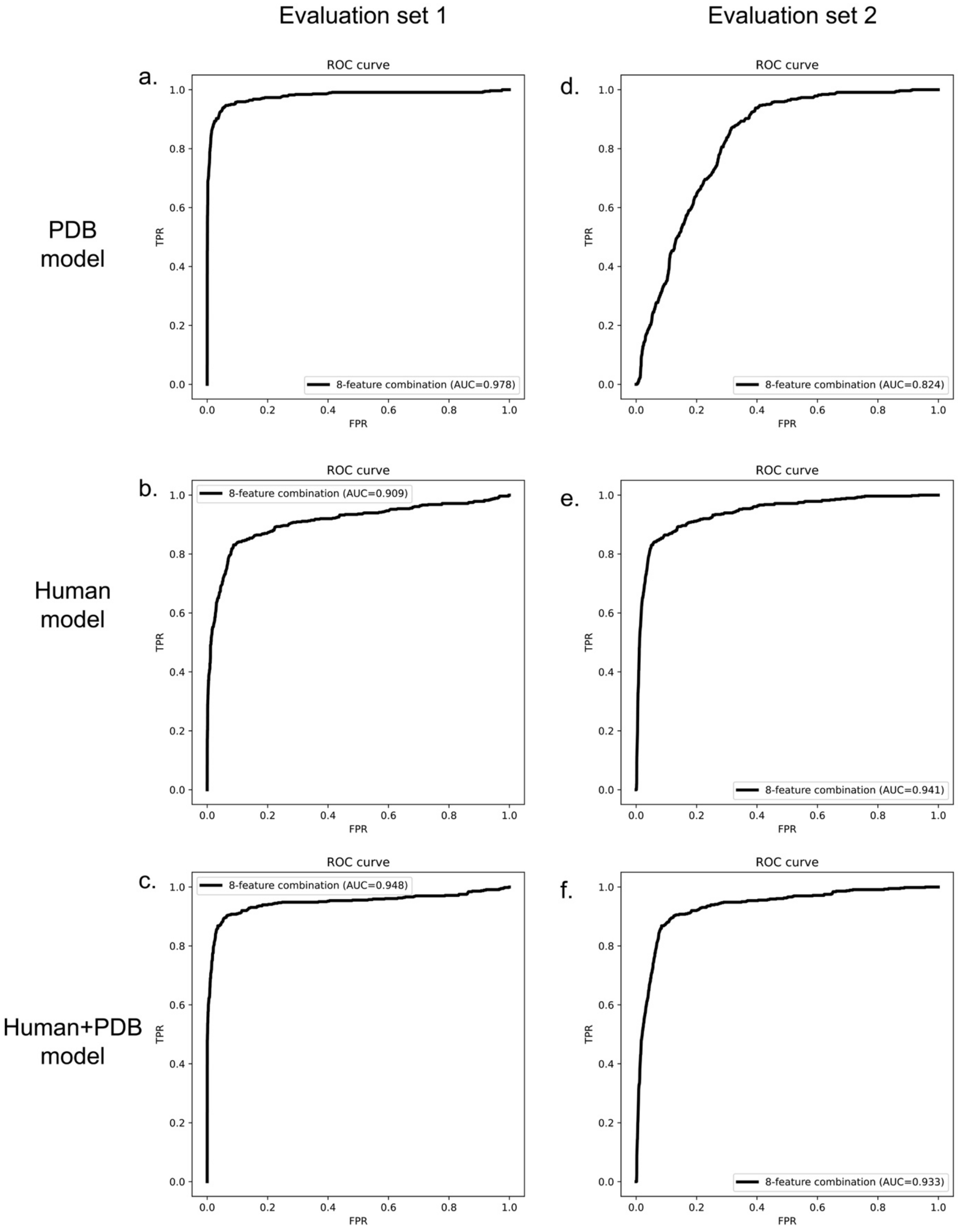

Figure S2. Final model performance via ROC curves, for 3 models. ROC curves on Evaluation set 1 (left) and Evaluation set 2 (right) for 3 different models: (a,d) PDB model, (b,e) Human model and (c,f) Human+PDB model.

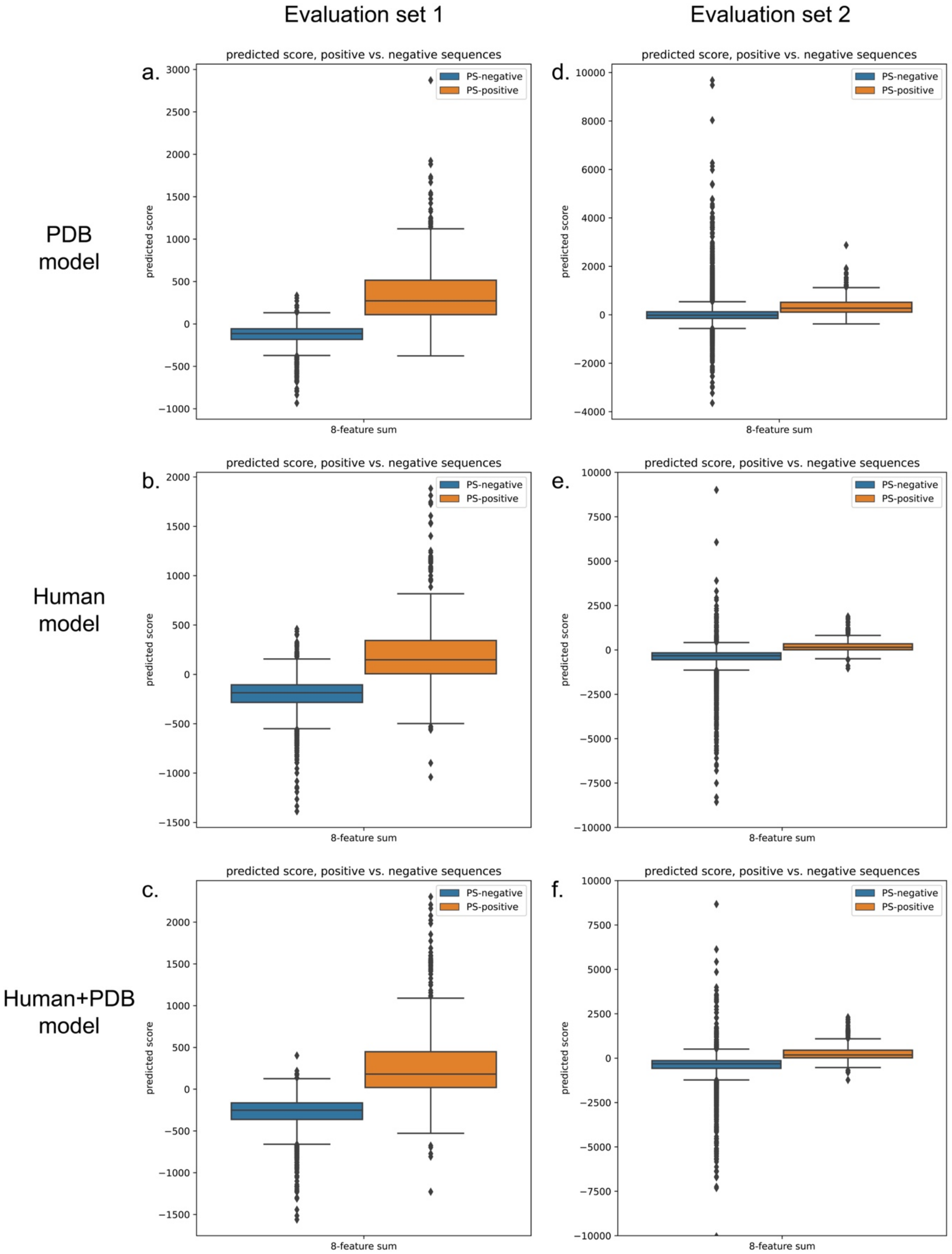

Figure S3. Final model performance via boxplots, for 3 models. Predicted score boxplots of positive vs. negative sequences on Evaluation set 1 (left) and Evaluation set 2 (right) for 3 different models: (a,d) PDB model, (b,e) Human model, and (c, f) Human+PDB model.

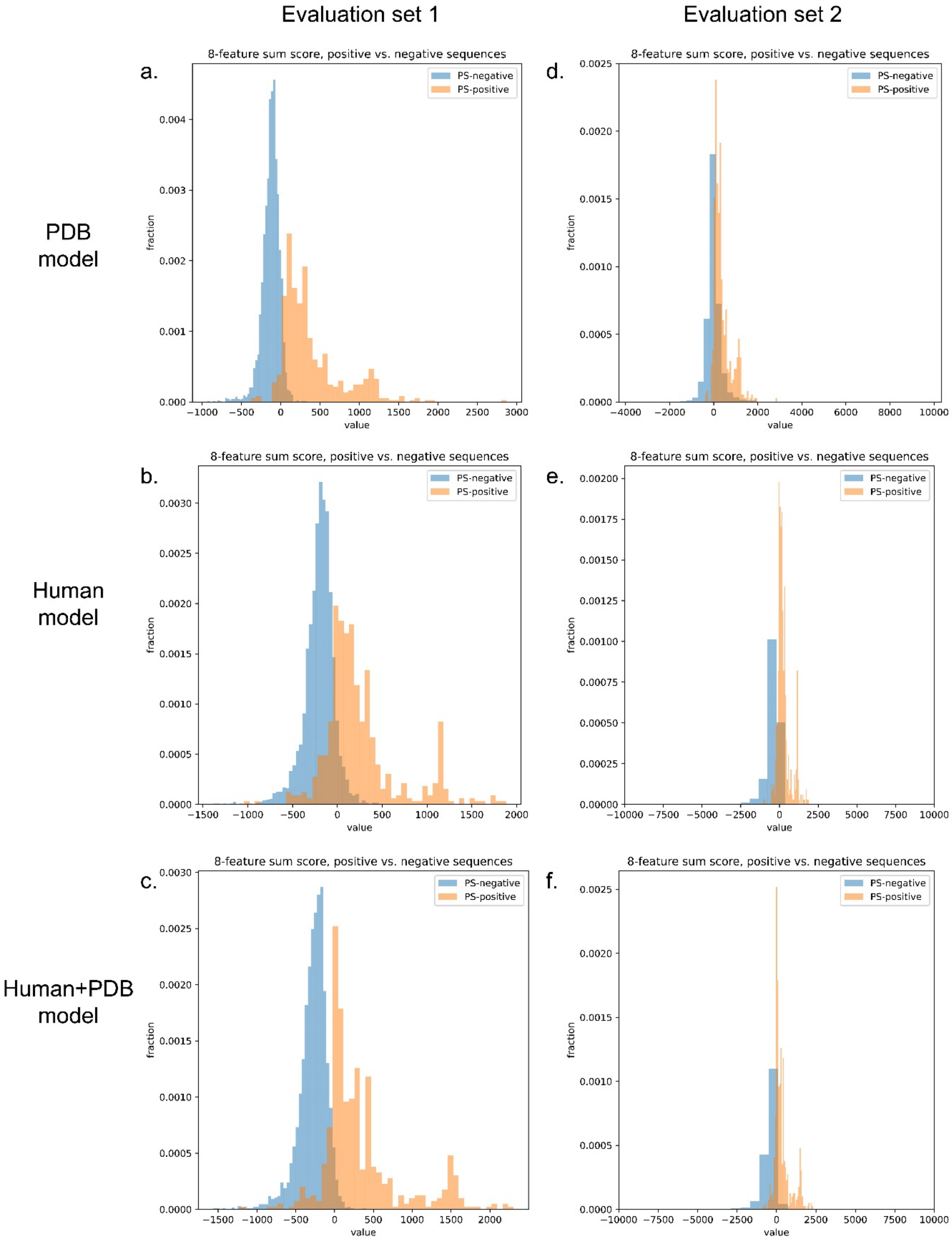

Figure S4. Final model performance via histograms, for 3 models. Distribution histograms of positive vs. negative sequences on Evaluation set 1 (left) and Evaluation set 2 (right) for 3 different models: (a,d) PDB model, (b,e) Human model, and (c,f) Human+PDB model.

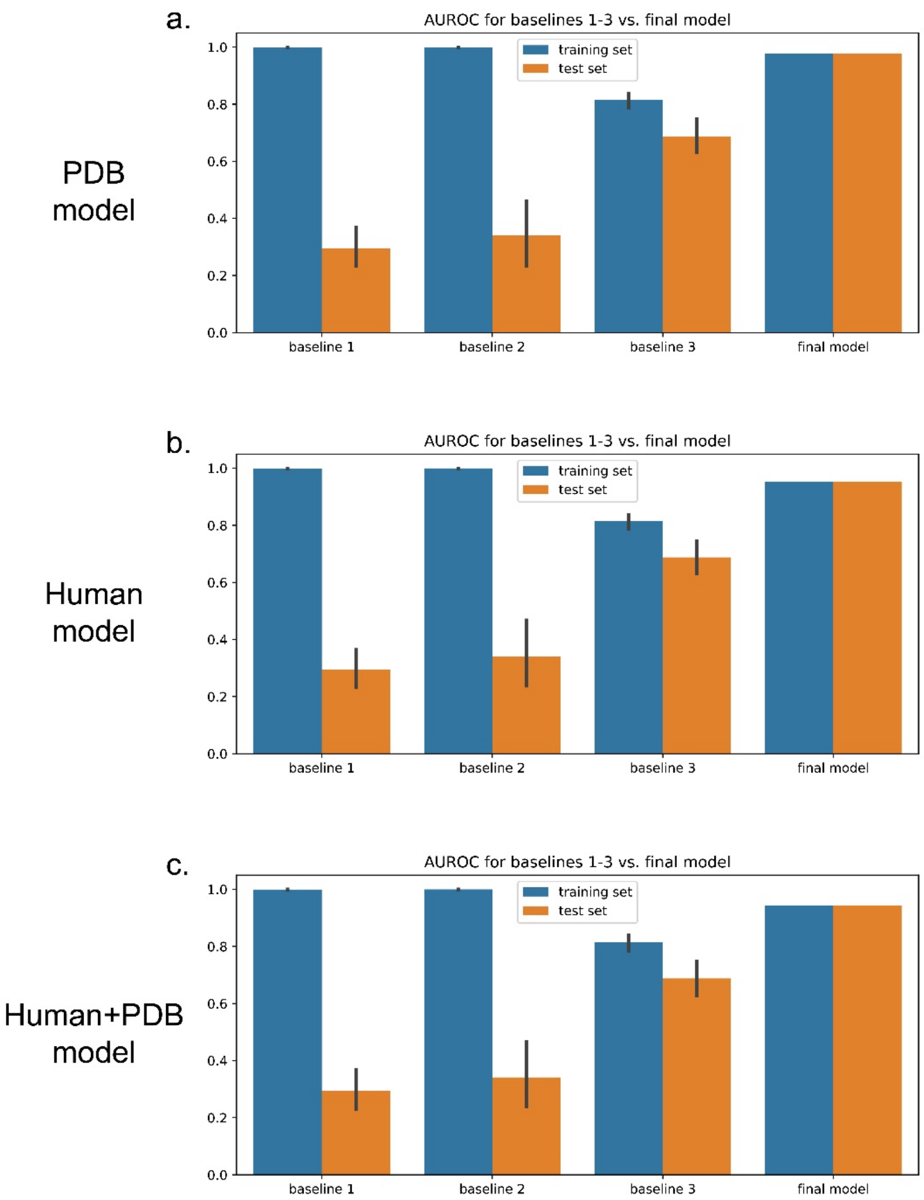

Figure S5. Comparison of three training baselines and the final predictor models for validation, for the 3 models. Performance comparisons are shown for (a) PDB model, (b) Human model, and (c) Human+PDB model. Baseline 1 was created by providing random values from a normal distribution N(0, 1) in the weight training step instead of providing PDB-based physical feature values into the genetic algorithm. Baseline 2 was created by providing random values from the distribution of residue-specific physical feature values instead of providing sequence-based physical feature values. Baseline 3 was created by optimizing 1 weight for 20 residue types for each physical feature (removing residue specificity) during training instead of optimizing 20 weights for 20 residue types for each physical feature.

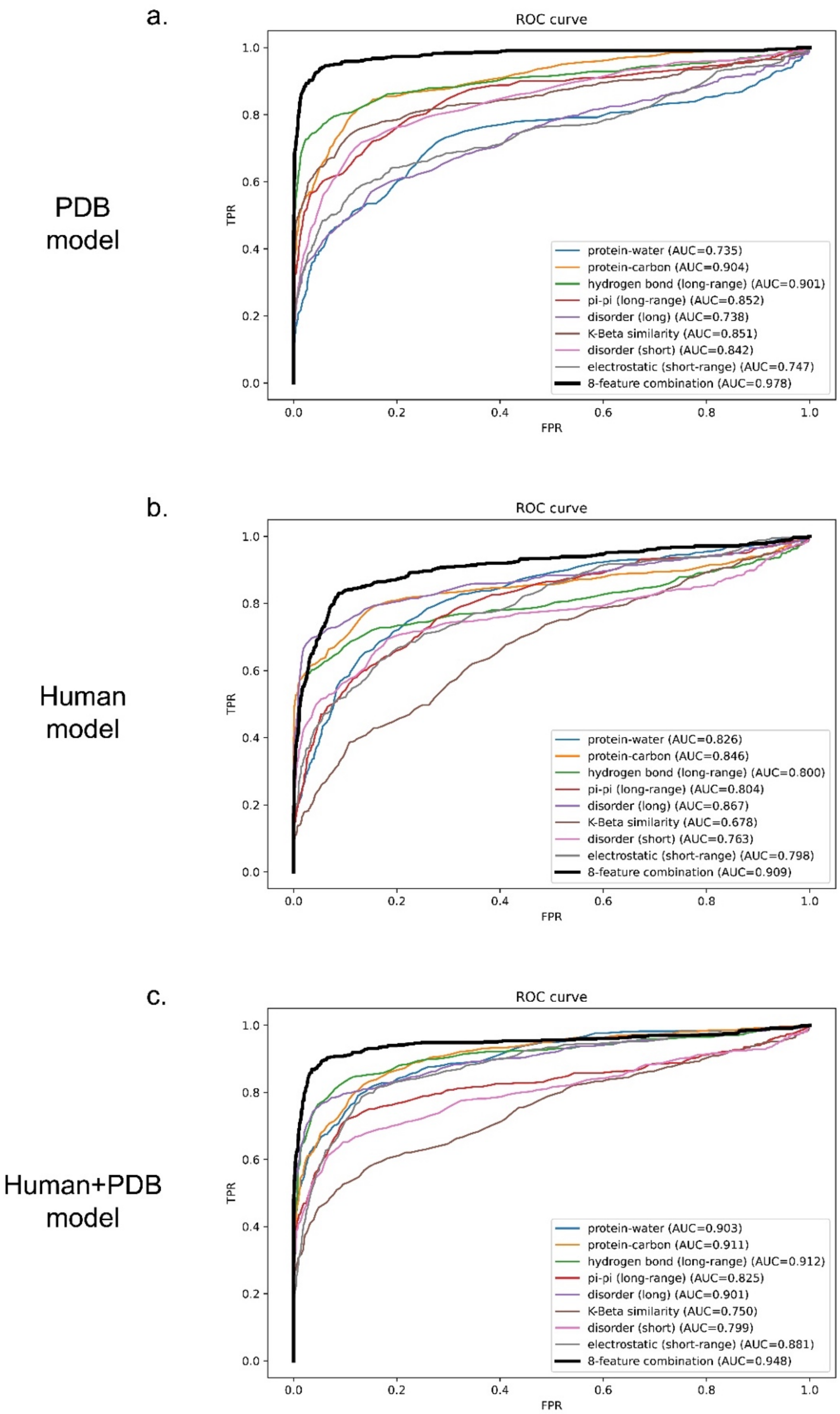

Figure S6. Comparison of performance via ROC curves of predictors trained on 8 features vs. 1 feature, for 3 models: (a) PDB model, (b) Human model, and (c) Human+PDB model.

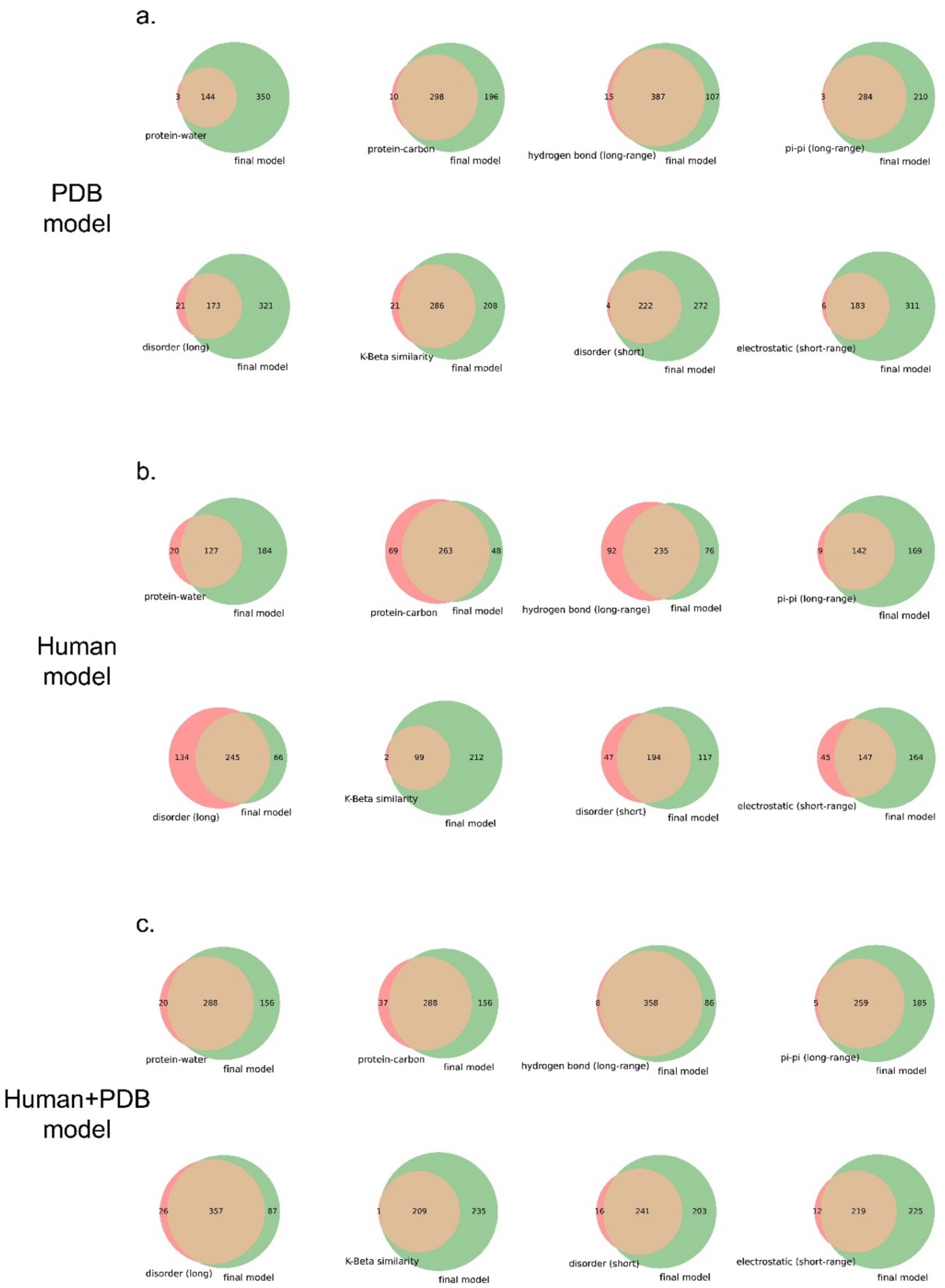

Figure S7. Comparison of performance via Venn diagrams of predictors trained on 8 features vs. 1 feature for the 3 models. Venn diagrams showing the coverage overlaps of PS-positive sequences by 1-feature predictors vs. the 8-feature predictor at a confidence threshold that returns 2% of the PDB, for 3 models: (a) PDB model, (b) Human model, and (c) Human+PDB model.

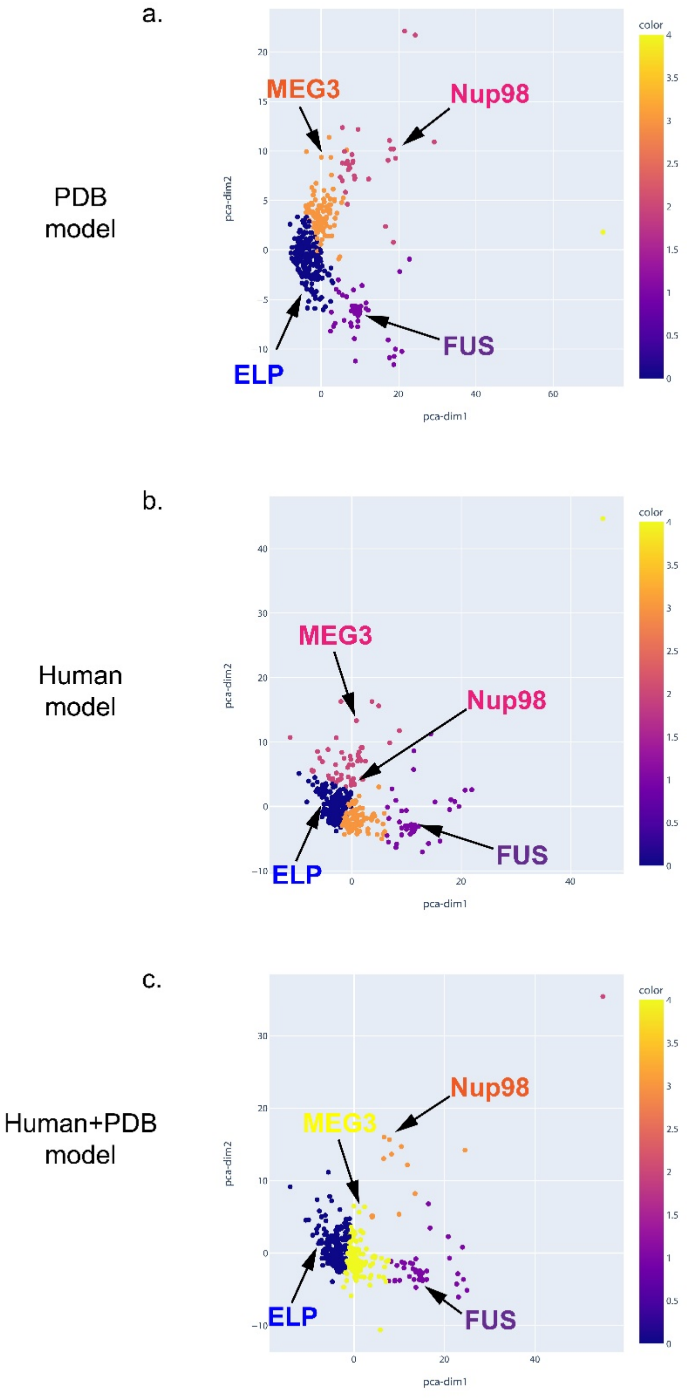

Figure S8. Feature score-based clustering for PS-positive proteins for the 3 models. Plots of 2 abstracted dimensions for clustering based on feature z-scores, showing the separation of different types of phase-separating sequences, for 3 models: (a) PDB model, (b) Human model, and (c) Human+PDB model.

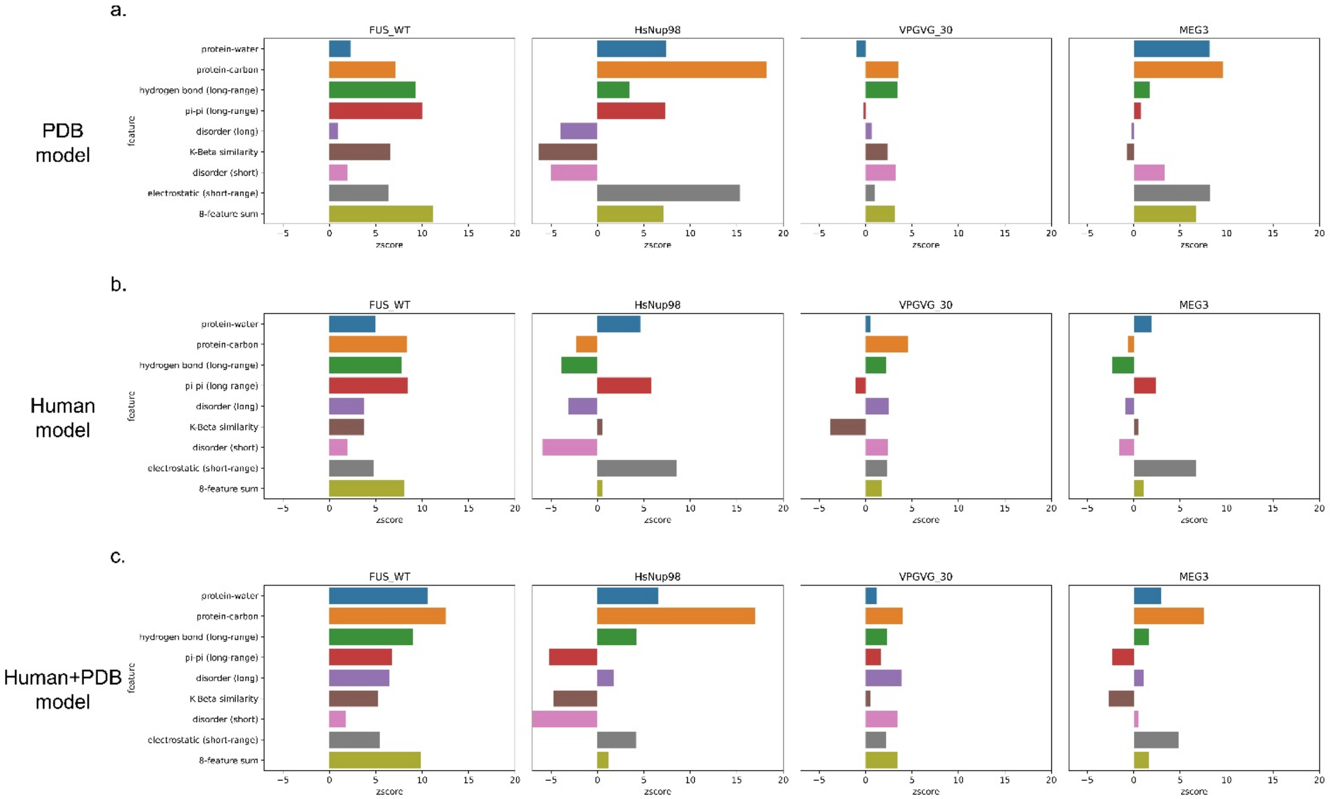

Figure S9. Feature score breakdown for example sequences from distinct clusters of PS-positive proteins for the 3 models. The score breakdown of 4 example sequences from 4 clusters in Fig S8 is shown for FUS (human), Nup98 (human), elastin-like peptide (ELP, VPGVG_30, 30 repeats of VPGVG) and MEG-3 (*C. elegans*) for 3 models: (a) PDB model, (b) Human model, and (c) Human+PDB model.

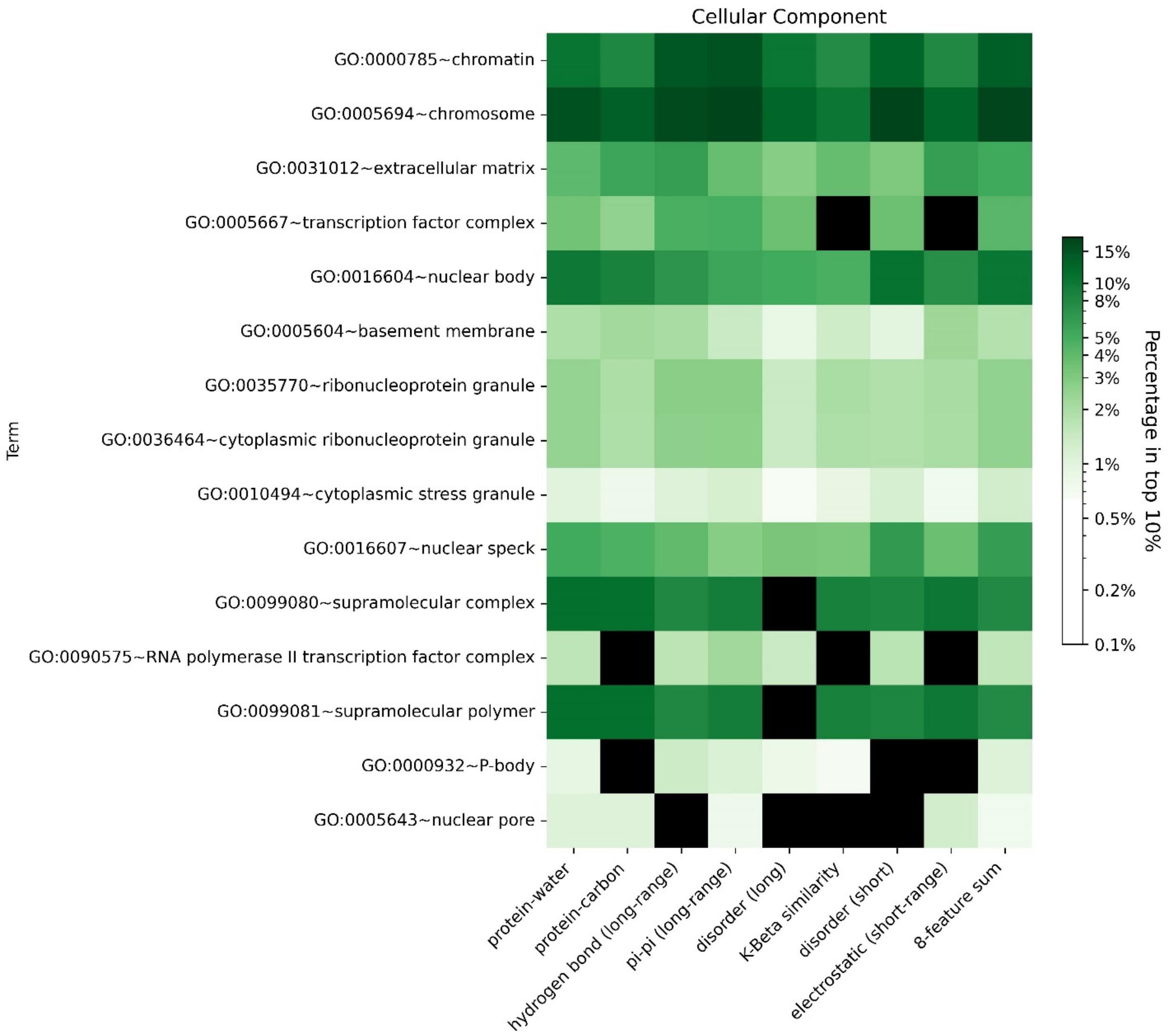

Figure S10. Enrichment heatmap by GO functional annotations for different features for the PDB model. Heatmap showing the enrichment of proteins with a given functional annotation that fall under a 10% confidence threshold for each single feature score and 8-feature sum score. The color gradient shows the natural logarithm of the enrichment percentage.

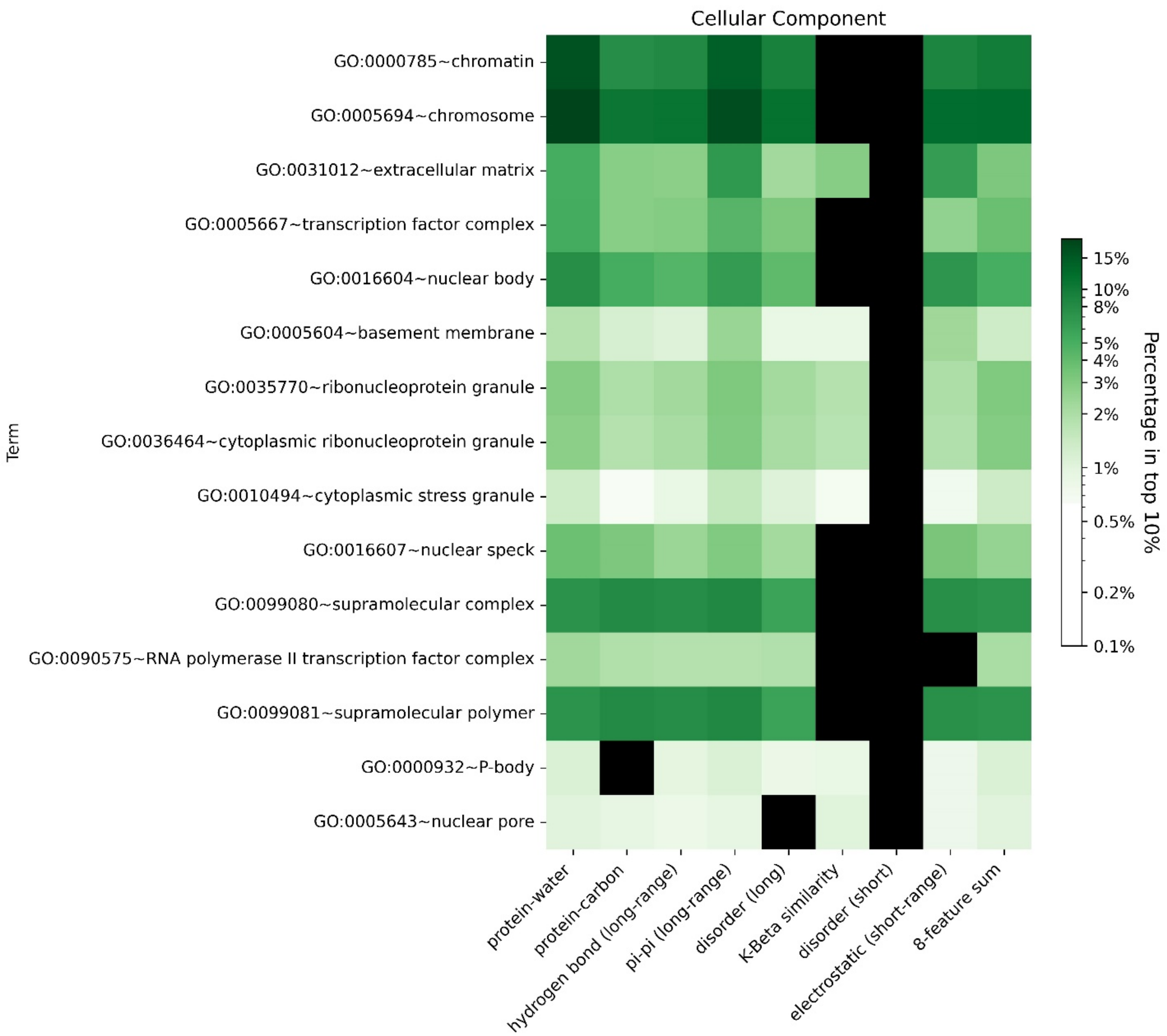

Figure S11. Enrichment heatmap by GO functional annotations for different features for the Human model. Heatmap showing the enrichment of proteins with a given functional annotation that fall under a 10% confidence threshold for each single feature score and 8-feature sum score. The color gradient shows the natural logarithm of the enrichment percentage.

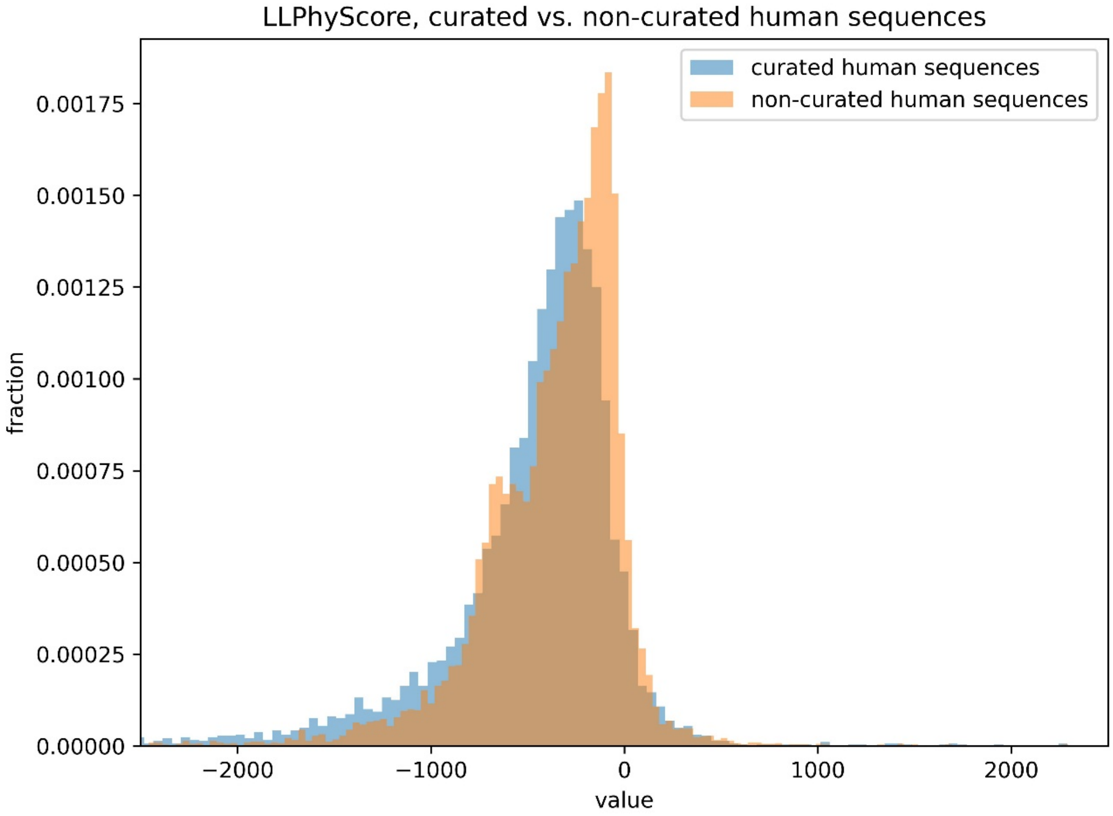

Figure S12. LLPhyScores for curated (CRAPome filtered) and uncurated human proteome sequences, demonstrating overall shift to negative scores for the curated sequences. A Z-test to quantify the statistical significance of the difference between to the scores for these two sets yielded a highly significant P-value of 5E-60.

Supplementary Tables S4-S7 are included in this Supplementary Material document here. **Supplementary Tables S1-S3 and S8-S11** are provided as separate files, described below.

Table S4. Constructed Training/Test/Evaluation sets and number of sequences in each set.

set type	set name	# positive samples	# negative samples
Training set	Training set 1	305	1703, PDB
	Training set 2	305	1703, Human
	Training set 3	305	1703, PDB + Human
Test set	Test set 1	260	1703, PDB
	Test set 2	260	1703, Human
	Test set 3	260	1703, PDB + Human
Evaluation set	Evaluation set 1	565	16794, PDB
	Evaluation set 2	565	20380, Human

Table S5. 16 physical features with corresponding definitions.

**Physical Feature**	**Definition**
pi-pi (short-range)	Pi-Pi contacts were defined using the method in Vernon et al. [1], and then divided into short range and long range by sequence separation. Less than 5 residues apart is defined as short range, and greater than or equal to 5 is defined as long range.
pi-pi (long-range)	
protein-water	Water and carbon counts were calculated only for the subset of proteins in our training set that have a total number of water molecules greater than the number of protein residues. This captures almost all models with resolution <= 1.8 but removes lower resolution models. Counts are measured for residues in their crystallographic context (measurement includes atoms from symmetry partners).
protein-carbon	
sec. structure (helices)	DSSP letter code was used for secondary structure assignments, with H/G used for helix, E for strand, and all others binned to loop.
sec. structure (strands)	
disorder (long)	For identifying disordered residues, a DSSP assignment of "not G/H/E" over a span of at least 3 residues was used to classify residues as loops. These loop residues were then assigned as short disorder if they fall within 3 residues of G/H/E and as long disorder if they do not.
disorder (short)	
electrostatic (short-range)	Phenix.reduce [2] was used to complete PDB structures by adding hydrogen atoms and charge interactions were calculated using the following pseudocode, with partial charges taken from the Talaris2014 energy function [3].<br><br>Q1 = partial_charge for atom X of amino acid 1<br>q2 = partial_charge for atom Y of amino acid 2<br><br>absF = 330.0 * abs(q1*q2)/(distance**2)<br>if q1*q2 < 0.0: absF *= -1.0<br><br>if SequenceSeparation >= 10: add absF to electrostatic (long range)<br>if SequenceSeparation < 10: add absF to electrostatic (short range)<br><br>Final per-residue values were then binned as follows:<br>bin = np.clip(int(round(residue_value / 16.0)), -9, 9)
electrostatic (long-range)	

<table>
<tr><td>hydrogen bond (short-range)</td><td rowspan="2">Structures probed for hydrogen OH-N hydrogen bonds using phenix [2], with the following commands used to extract hydrogen bond information.
phenix.reduce -Quiet -FLIP [pdb file] > ./PHENIX_ALL/PHENIXL.pdb
phenix.probe "NITROGEN,OXYGEN,HYDROGEN" -Quiet -ONEDOTeach -NOCLASHOUT -SUMMARY -NOVDWOUT ./PHENIX_ALL/PHENIXL.pdb | grep greentint > ./N17.PHENIX/HLIST.'+pdb

Bonds were than classified as short range and long range by sequence separation (short range < 5, long range $\geqslant$ 5).</td></tr>
<tr><td>hydrogen bond (long-range)</td></tr>
<tr><td>cation-pi (short-range)</td><td rowspan="2">We reran the electrostatic scores after adding arbitrary partial charges to the surfaces of aromatic rings, with a partial charge value of -0.05 added 0.85 Å above and below the plane of the ring for each atom, counterbalanced by a partial charge of 0.1 at the atom.

The cation pi-score is then taken from the difference between this modified score and the unmodified electrostatic score.</td></tr>
<tr><td>cation-pi (long-range)</td></tr>
<tr><td>K-Beta similarity</td><td rowspan="2">Superpositions to kinked beta fibrils were made for chain A in each of 5 structures, PDB IDs 6bwz, 6bxv, 6bxx, 6bzm, and 6bzp. The full chain of each was superimposed to every overlapping window (same number of residues as the chain with none missing) in our PDB training set and kinked beta similarity was measured for each individual PDB residue by taking the minimum CA-RMSD over all measurements the residue was involved in.

Residues were then classified as K-Beta similar if the minimum CA-RMSD was under 1.0 Å and as K-Beta dissimilar if it was over 2.0 Å.</td></tr>
<tr><td>K-Beta non-similarity</td></tr>
</table>

Table S6. The “signs” of 16 features determined by AUROC direction during individual feature training. Feature with a positive correlation to phase separation prediction are in black and those with a negative correlation to phase separation prediction are in red.

feature name	initial AUROC	final AUROC	feature sign
pi-pi (short-range)	0.713	0.952	+
pi-pi (long-range)	0.834	0.968	+
protein-water	0.806	0.966	+
protein-carbon	0.155	0.035	-
sec. structure (helices)	0.240	0.054	-
sec. structure (strands)	0.787	0.949	+
disorder (long)	0.904	0.961	+
disorder (short)	0.691	0.954	+
electrostatic (short-range)	0.854	0.959	+
electrostatic (long-range)	0.263	0.048	-
hydrogen bond (short-range)	0.696	0.955	+
hydrogen bond (long-range)	0.794	0.967	+
cation-pi (short-range)	0.355	0.039	-
cation-pi (long-range)	0.226	0.033	-
K-Beta similarity	0.833	0.938	+
K-Beta non-similarity	0.239	0.042	-

Table S7. The AUROC of different trained feature combinations.

feature combination	AUROC (training set)	AUROC (test set)
16 features	0.959	0.935
12 features	0.962	0.933
final model (8 features)	0.969	0.942
final model (8 features) trained on training set 1+ test set 1 (“PDB model”), using Evaluation set 1	0.978	
final model (8 features) trained on training set 1+ test set 1 (“PDB model”), using Evaluation set 2	0.824	
final model (8 features) trained on training set 2 + test set 2 (“Human model”), using Evaluation set 1	0.908	
final model (8 features) trained on training set 2 + test set 2 (“Human model”), using Evaluation set 2	0.941	
final model (8 features) trained on training set 3+ test set 3 (“Human + PDB model”), using Evaluation set 1	0.947	
final model (8 features) trained on training set 3+ test set 3 (“Human + PDB model”), using Evaluation set 2	0.933	

Separate Supplementary Files

One attached Excel file contains, on separate tabs, **Tables S1-S3 and S8-S11**. **Table S1**. Detailed information of 565 PS-positive sequences with PMID of each sequence's paper.
Table S2. Uniprot IDs of 6102 sequences from human proteome that represent the negative training set using CRAPome as filtering method.
Table S3. LLPhyScore and CRAPome scores for all human sequences, including both those within the curated negative training set and those not in the curated list.
Table S8A. GO enrichment analysis for PDB+Human model.
Table S8B. GO enrichment analysis for PDB model.
Table S8C. GO enrichment analysis for Human model.
Table S9. Uniprot IDs of 3406 sequences from PDB base.
Table S10. Uniprot IDs of 3406 sequences randomly selected from Human base in Table S2.
Table S11. Uniprot IDs of 6812 sequences from PDB+Human base.

File S1. 565 PS-positive sequences (fasta file).
File S2. Hierarchical clustering dendrogram of PS-positive sequences (jpeg file).
File S3. 16794 PDB sequences (fasta file).
File S4. 20380 Human sequences (fasta file).

Supplementary Material References

1 Vernon, R. M. et al. Pi-Pi contacts are an overlooked protein feature relevant to phase separation. eLife 7, e31486, doi:10.7554/eLife.31486 (2018).
2 Adams, P. D. et al. PHENIX: a comprehensive Python-based system for macromolecular structure solution. Acta Crystallographica Section D: Biological Crystallography 66, 213-221 (2010).
3 O'Meara, M. J. et al. Combined covalent-electrostatic model of hydrogen bonding improves structure prediction with Rosetta. Journal of chemical theory and computation 11, 609-622 (2015).
