# Supplementary figures and images for: An Interpretable Machine-Learning Algorithm to Predict Disordered Protein Phase Separation Based on Biophysical Interactions
Source: Biomolecules. 2022 Aug 17;12(8):1131. doi: 10.3390/biom12081131 (PMC9405563; doi:10.3390/biom12081131)

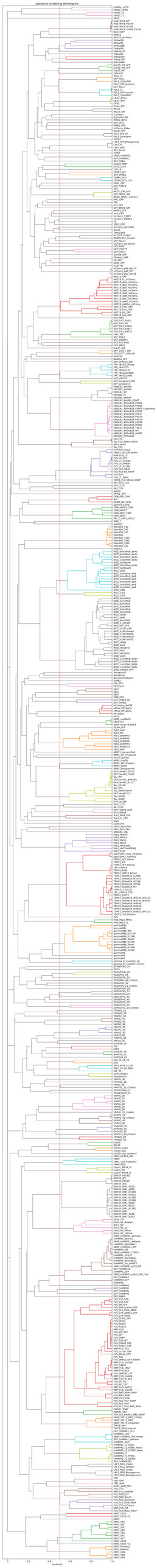

Supplement: Supplementary file 1 [file biomolecules-12-01131-s001.zip › File_S2_sequence_clustering_dendrogram_vertical.pdf]
